# Effect of Returnable Material in Batch on Hot Tearing Tendency of AlSi9Cu3 Alloy

**DOI:** 10.3390/ma14071583

**Published:** 2021-03-24

**Authors:** Justyna Kasińska, Marek Matejka, Dana Bolibruchová, Michal Kuriš, Lukáš Širanec

**Affiliations:** 1 Department of Metal Science and Materials Technology, Kielce University of Technology, Al. Tysiąclecia Państwa Polskiego 7, 25 314 Kielce, Poland; kasinska@tu.kielce.pl; 2 Department of Technological Engineering, Faculty of Mechanical Engineering, University of Zilina, Univerzitná 8215/1, 010 26 Žilina, Slovakia; danka.bolibruchova@fstroj.uniza.sk (D.B.); michal.kuris@fstroj.uniza.sk (M.K.); lukas.siranec@fstroj.uniza.sk (L.Š.)

**Keywords:** Al-Si-Cu alloy, returnable material, hot tearing susceptibility, hot tearing index, complex geometry castings, microstructure

## Abstract

The main reason for the use of returnable material, or recycled alloys, is a cost reduction while maintaining the final properties of the casting. The casting resulting quality is directly related to the correct ratio of commercial grade alloy and alloy made by remelting the returnable material in the batch. The casting quality is also affected by the purity of the secondary raw materials used, the shape complexity and the use of the casting itself. The presented article focuses on the effect of increasing the returnable material content in the batch on the hot tearing susceptibility of AlSi9Cu3 alloy. Hot tears are a complex phenomenon that combines metallurgical and thermo-mechanical interactions of the cast metal. Hot tearing susceptibility was evaluated on the basis of quantitative (HTS—hot tearing susceptibility index) and qualitative evaluation. The negative effect of returnable material in the batch was already manifested at a 20% content in the batch. The critical proportion of the returnable alloy in the batch can be stated as 50%. The alloy with a 50% returnable material content manifested insufficient results of the HTS index and qualitative evaluation, which means increased sensitivity to tearing. The negative effect of returnable material and the increased sensitivity were also confirmed in the evaluation of the fracture surface and hot tear profile. The microstructure of alloys with 50% and higher proportion of returnable material was characterized by a higher amount of iron phases (mainly Al_5_FeSi), whose sharp ends acted as critical regions of hot tearing and subsequent hot tear propagation, which had a major impact on the increase in hot tearing susceptibility.

## 1. Introduction

At present, we can talk about almost 100% recycling of all aluminum products. The recycling process continues the life of aluminum and its alloys. Approximately 70% of recycled aluminum alloys are subsequently used for castings intended mainly for the automotive industry. The recycling process as well as the replacement of primary alloys with remelted returnable material allows foundries to be more efficient in terms of raw materials and energy. For this reason, we can now encounter castings made by exclusively using returnable material. These castings are able to meet the quality requirements associated with their demanding specifications. However, the overall requirements for melt quality vary and are related to the shape complexity and purpose of the casting. For highly stressed or castings with complex geometry, it is necessary to find the ideal ratio of primary alloy and remelted returnable material in the batch, and to determine the right compromise between the quality and price of the casting [1,2,3,4,5,6].

The presence of hot tears deteriorates the mechanical and utility properties of the casting. The result of hot tearing and hot tear propagation is a breach of the casting integrity (surface or internal) during its solidification. Ultimately, a hot tear can manifest itself as an unrecoverable fault, which in turn leads to component disposal and economic and time losses. Minimizing the hot tear formation and subsequent losses can be prevented by several options, such as the appropriate choice of material used, mold design and optimization of the gating system [7,8,9,10,11,12].

Verö has conducted research on the formation of hot tears in aluminum alloys. He found that the contraction of the primary crystals during solidification creates stress that causes the formation of hot tears. In the first phase of solidification, hot tears do not occur due to the non-formation of a continuous matrix. In the next phase, dendrites grow and contact each other, forming a continuous matrix. Continued solidification by its effect generates stress when the contraction of the solidifying alloy is limited by the core or mold. Later, a theory of hot tear formation based on the accumulation of deformation energy was presented. The theory was based on the condition that the deformation energy primarily controls the hot tear formation process. The study is based on the concept of the presence of a layer of liquid metal, and at the same time, proves that if a critical amount of deformation energy accumulates in the heat node, hot tears are formed [13]. Using this knowledge, Campbell then mathematically expressed deformation energy in the heat node through the following equation:

ε = (α × ΔT × L)/l [Pa],(1)

where α—coefficient of thermal expansion (K−1), ΔT—length of the two-phase zone (mm), L—length of the casting (mm) and l—length of the heat node (mm). It is clear from Equation (1) that the deformation energy can be minimized by removing heat nodes, inoculating and reducing temperature differences during solidification of the casting [14]. Several factors act on the formation of hot tears. The main factors include the effect of chemical composition, casting temperature and mold temperature, and the design of the mold. 

### 1.1. Chemical Composition

Chemical composition has a fundamental impact on the resulting susceptibility of the alloy to hot tearing, whether it is individual elements or the width of the solidification interval, which is directly derived from the chemical composition [15,16]. In general, the wider the solidification interval, the higher the alloy susceptibility to hot tearing.

However, in the case of Al-Si-based alloys, it applies that after exceeding a certain critical proportion of silicon (approximately 5 wt.%), the resulting eutectic content is already sufficient, despite the fact that the solidification interval is relatively wide and can “surround” primary dendrites and thus rapidly increase the ability to replenish the melt to critical regions in order to compensate for emerging hot tears. Lin confirmed in his experiments that in the case of silicon, the width of the solidification interval is not directly proportional to the hot tearing susceptibility rate, and therefore other factors must be taken into account, which affect the formation or suppression of hot tears [17,18].

In the Al-Si-Cu alloy, copper has a significant effect on the sensitivity to hot tearing, by influencing the solidification interval. Higher concentrations of Cu extend the solidification interval and thus cause a reduction in resistance to hot tear formation. In general, the lowest resistance to hot tearing corresponds to the amount of Cu, which corresponds to the maximum width of the interval [19].

For certain iron content, and with increasing silicon content, the temperature and time decrease at which the iron-rich phase particles may form prior to the Al-Si eutectic. According to Taylor, the negative effect of iron on hot tear formation is caused by a growing iron content, which increases the number and size of Fe-based phases, which are directly involved in the fracture mechanism. Large plates of the iron phase β can restrain the flow of the melt into the inter-dendritic spaces, thus suspending the deposition of the melt in critical sites and promoting the formation of hot tears and their further propagation [20].

The detrimental effect of intermetallic particles on hot tear formation is attributed to their much greater susceptibility to fracture under tensile loads. After the aluminum matrix solidifies, the total stresses in the system increase. As a critical point of microtears formation or as a stress concentrator, becomes the sharp end of the needles of the iron phase oriented perpendicular to the load axis. Due to the fragility of the phase, conditions are created for hot tear propagation along the phase needle [21].

### 1.2. Mold Temperature

The effect of mold temperature on hot tear formation is mainly related to influencing the cooling rate of the casting during solidification. Influencing the cooling rate affects mechanical properties, microstructure and also the occurrence of hot tears. In general, hot tearing tendency decreases with increasing mold temperature. The reason for the reduction in hot tearing susceptibility can be attributed to the ability to better replenish the melt to critical regions during solidification at higher mold temperatures [22,23].

### 1.3. Casting Temperature

While the results are unambiguous regarding the effect of chemical composition and mold temperature on hot tear formation, they differ regarding the casting temperature. These conflicting views may be due to the following factors. A higher temperature of the melt widens the regions of heat nodes, which can reduce the hot tearing tendency, but it also prolongs the life of the liquid metal layer, thus increasing hot tearing susceptibility. It is likely that elevated melt temperatures cause higher temperature drops during solidification, resulting in the growth of negative columnar grains. In general, alloys with such a structure are more susceptible to hot tearing than alloys with irregular grains. Some research has confirmed that the effect of casting temperature varies depending on the hot tearing detection method applied [24,25,26,27].

## 2. Materials and Methods

### 2.1. Material AlSi9Cu3

Hot tearing susceptibility evaluation was performed on a hypo-eutectic AlSi9Cu3 alloy (A226, EN AC—46000). A dominant amount of AlSi9Cu3 alloy castings are used in the automotive and electrical engineering industries. AlSi9Cu3 alloy castings are characterized by good mechanical properties, strength at elevated temperatures, as well as good running property and low shrinking tendency. The chemical composition of the experimental alloy AlSi9Cu3 (commercial purity—ingots purchased from company Dor, Považská Bystrica, Slovakia) is given in Table 1.

### 2.2. Newly Formed Experimental AlSi9Cu3 Alloys

In the first step of the experimental part, a “new” AlSi9Cu3 alloy was created by remelting the foundry returnable material (ingot residues, remains of gating systems and risers). An electric resistance furnace was used to remelt the foundry returnable material. The batch (with a total weight of 95 kg) was melted in a steel crucible with a volume of 100 kg, to which a graphite protective coating was applied before melting. Subsequently, the foundry returnable material was cast as ingots into pre-prepared metal molds (with a temperature of 150 ± 5 °C).

In the next step of the experimental part, foundry returnable material ingots together with a commercial grade alloy were used to cast five experimental AlSi9Cu3 alloys. The alloys were cast with different contents of returnable material and the commercial grade alloy in the batch. The total weight of the batch was 12.5 kg. Melting was performed in an electric resistance furnace (LAC, Židlochovice, Czech Republic) with a T15 type regulator with a capacity of 15 kg in a graphite crucible, which was treated with a protective coating. The casting temperature was 750 ± 5 °C. The alloys were designated R-20, R-50, R-70, R-80 and R-90, where the number indicates the percentage of returnable material in the batch (R-20 = 20% returnable alloy). Table 2 shows the chemical composition of the AlSi9Cu3 alloy cast from foundry returnable material and five alloys with an increasing content of returnable material. The chemical composition was determined by arc spark spectroscopy (Bunker—Q2 ION, Kalkar, Germany).

### 2.3. Method for Hot Tearing Susceptibility Evaluation and the Equipment

Hot tearing susceptibility of AlSi9Cu3 alloy with different contents of returnable material in the batch was evaluated based on two criteria. The first criterion assessed was quantitative evaluation of the so-called “hot tearing index” (HTI) and the second criterion was qualitative assessment. The AlSi9Cu3 commercial grade alloy was evaluated in the experiment together with five experimental alloys.

The measuring apparatus and the mold used for the experimental process were designed and manufactured at the Department of Technological Engineering of the University of Žilina. The mold uniquely enables the concurrent quantitative as well as qualitative evaluation of hot tearing susceptibility.

**Figure 1:**
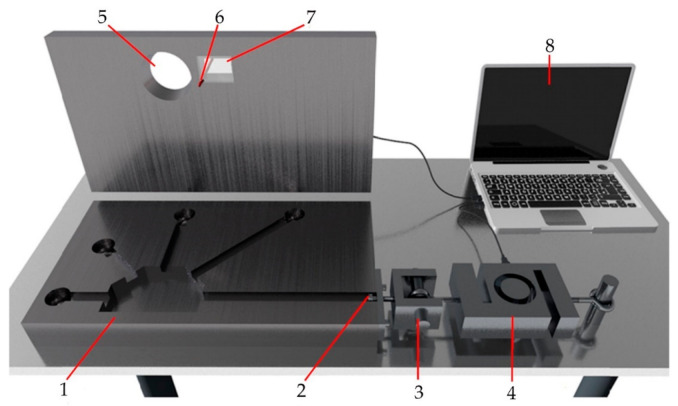
Measuring apparatus, 1—mold, 2—anchoring screw, 3—gripping mechanism, 4—load cell, 5—inlet, 6—thermocouple, 7—refractory glass, 8—data processing.

The mold consists of a simple gating system, from which five arms of different lengths emerge. Four arms are anchored at the end, which will support shrinkage effect, consequently leading to hot tearing susceptibility. The four anchored arms are intended for qualitative evaluation (i.e., hot tearing index) and their lengths are given in Table 3. The fifth arm ends with an anchoring screw and was used for qualitative evaluation. Figure 1 schematically shows the individual parts of the device used in the experimental process. Each alloy was cast four times. The casting was removed from the mold after five minutes to visually evaluate the hot tearing index. The melt temperature during casting was 750 ± 5 °C and the mold was preheated to 150 ± 10 °C.

#### 2.3.1. Quantitative Evaluation—Hot Tearing Index (HTI)

Two versions of evaluation (equations determining HTI) and their combination were used to evaluate HTI. In the first version of the equation expressing HTI_1_ [16], the numerical value of HTI_1_ depends on the nature and size of the hot tear (“weighting factor”—WF), the number of tears (NOT) and the number of evaluated arms (amount of cast samples) (ACS):

HTI_1_ = (NOT × ∑ WF)/ASC (2)

In the second version of the equation [28,29,30,31], the HTI_2_ value depends on the hot tear position on the test arm (“tear position”—TP), the length of the test arm (“arm length”—AL) and on the nature and size of the hot tear (“weighting factor”—WF). HTI_2_ is defined as:

HTI_2_ = ∑ WF × TP × AL (3)

**Figure 2:**
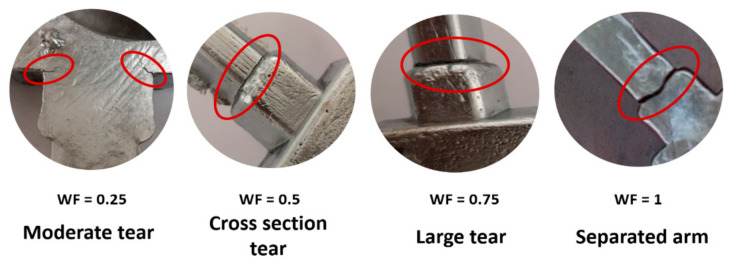
Weighting factor categories.

The hot tear coefficient WF (weighting factor) in both versions indicates the degree of hot tear severity (nature and size). The WF value is divided into four categories, each of which is assigned a different numerical value. Hot tear severity gradation and numerical values are shown in Figure 2. Table 3 shows the numerical values of the coefficients for the arm length (AL) and the hot tear position on the test arm (TP).

The resulting numerical HTI values can be characterized as a measure of the susceptibility to hot tearing, the so-called “hot tearing susceptibility” (HTS) (Table 4).

The third version of the equation to determine the value of hot tearing susceptibility arises from a combination of the two previous equations. Substituting the equation HTI_2_, which more closely characterizes the formed hot tear in the equation HTI_1_, a value is obtained taking into account the nature of both equations, and thus a more complex view. HTI_3_ can be defined as:

HTI_3_ = (NOT × ∑ (WF × TP × AL))/ASC, (4)

#### 2.3.2. Qualitative Evaluation of Test Samples

Qualitative evaluation was performed by sequential analysis of the strength and temperature curves of the given alloy recorded during the casting process by means of a K-type thermocouple (NiCr-Ni) and a through-arm (the longest, fifth arm). The arm was connected to a force transducer (S9M Force Transducer, HBM, Darmstadt, Germany) (load cell) using an anchoring screw. The values were recorded in LabView 2 Hz software (version 18.5, National Instruments, Austin, TX, USA). Graphs were created from the measured values, which were subjected to analysis in terms of the tensile force magnitude and the course of the curve. Using the first derivative of the force, we obtained a curve of the load increase rate.

#### 2.3.3. Metallographic Evaluation Equipment

Fracture surfaces and hot tear profiles of the experimental material were evaluated using a NEOPHOT 32 optical microscope (OM) and scanning electron microscope (SEM) observations with EDX (energy dispersive X-ray) analysis using a VEGA LMU II scanning electron microscope connected to energy dispersive X-ray spectroscopy (Brucker Quantax EDX analyzer, Bunker, Kalkar, Germany). Metallographic evaluations were performed on samples, the preparation of which consisted of coarse and fine wet grinding, polishing on an automatic instrument using a diamond emulsion, and etching (0.5% HF solution). Samples for the evaluation of the profile of hot tears and fracture surfaces were taken from the arms for qualitative evaluation. We used those arms where the arm was completely torn off (R-50, R-70 and R-90 alloys).

## 3. Results

### 3.1. Quantitative Evaluation—Hot Tearing Index (HTI) 

**Figure 3:**
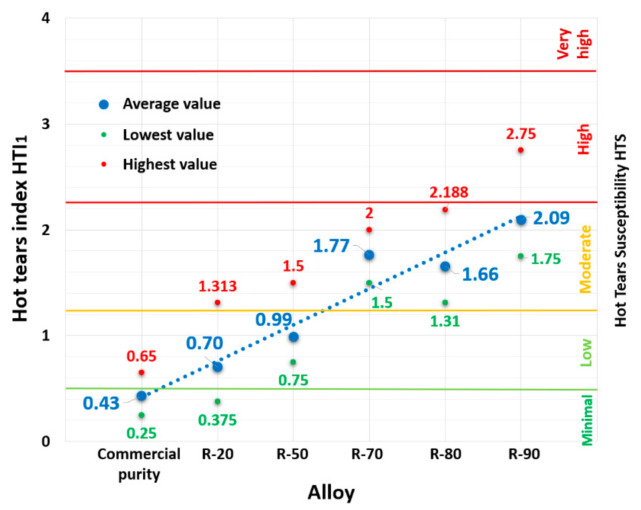
Dependence of hot tears susceptibility to AlSi9Cu3 alloy according to HTI_1_.

It can be seen from the results shown in the graph for HTI_1_ (2) (Figure 3) that the reference alloy consisting only of the commercial grade AlSi9Cu3 alloy reached an HTI_1_ value of 0.43, which means a minimum susceptibility to hot tearing. After increasing the content of returnable material in the batch to 20% and 50%, the susceptibility to hot tearing increased from the minimum value to a low HTS level. The R-20 alloy reached HTI1 = 0.70, and the R-50 alloy had HTI_1_ = 0.99. In the case of alloys consisting of a major proportion of returnable material, the resulting HTS values shifted to a slight rate of hot tearing susceptibility. The maximum value of the HTI_1_ index of 2.09 was recorded for the R-90 alloy, which represents an approximately 5-fold increase compared to the commercial grade alloy (the reference alloy).

**Figure 4:**
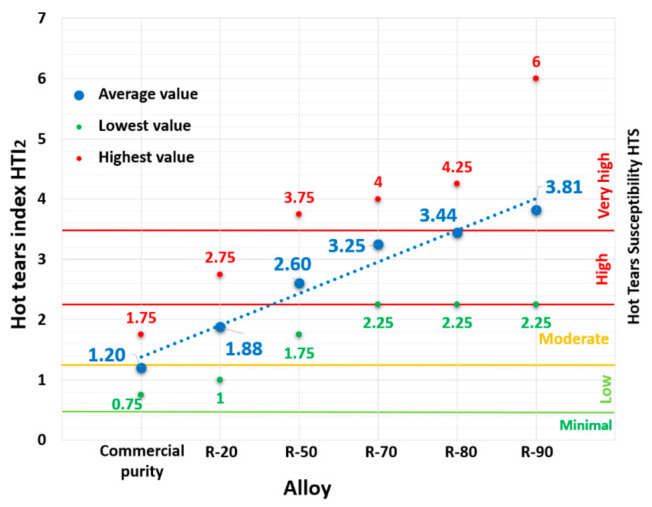
Dependence of hot tears susceptibility of AlSi9Cu3 alloy according to HTI_2_.

By using the second Equation (3) for HTI_2_, the numerical value of which depends not only on the nature of the hot tear but also on its position and the arm length, we obtained orders-of-magnitude higher values of the HTI_2_ index compared to HTI_1_. The resulting values of HTI_2_ are shown graphically in Figure 4. The HTS index of the reference alloy in this case was on the border between low and moderate hot tearing susceptibility with a HTI_2_ value of 1.2. Compared to HTI_1_, where a high level of hot tearing susceptibility was not reached by any alloy, then, using Equation (2) for HTI_2_, the R-70 and R-80 alloys reached this level. The R-90 alloy reached a very high hot tearing susceptibility HTI_2_ value of 3.81.

**Figure 5:**
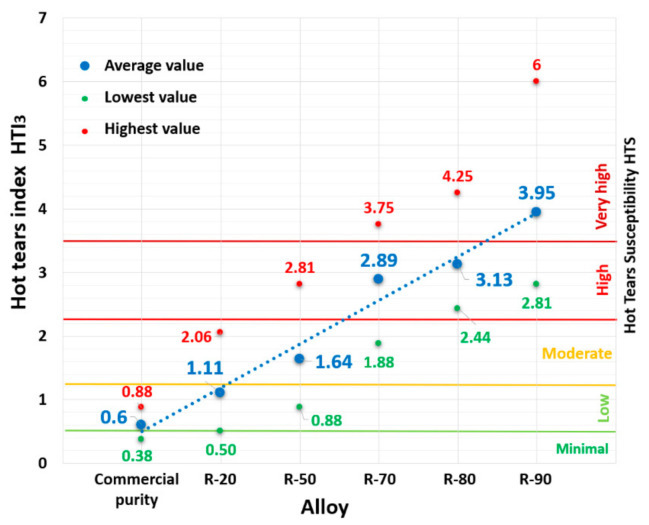
Dependence of hot tears susceptibility of AlSi9Cu3 alloy according to HTI_3_.

The graph in Figure 5 shows the HTI_3_ values obtained from Equation (4). Using the HTI_3_ relationship, the largest difference between the reference alloy and the R-90 alloy (containing 90% returnable material) was measured. The reference alloy with an HTI_3_ of 0.60 showed low susceptibility to hot tear formation. The R-20 alloy also reached a low susceptibility level. On the other hand, in the R-90 alloy, the HTI_3_ was 3.95, and thus the alloy reached very high hot tearing susceptibility.

### 3.2. Qualitative Evaluation of Test Samples

**Figure 6:**
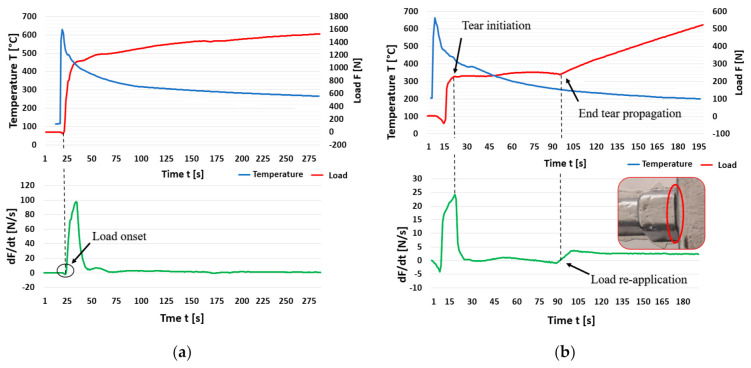
Curve of load force, temperature and load force ratio: (**a**) AlSi9Cu3 Commercial purity (fourth measurement), and (**b**) alloy R-50 (second measurement).

**Figure 7:**
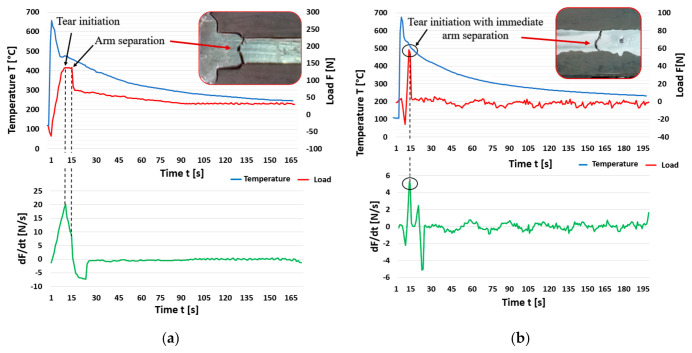
Curve of load force, temperature and load force ratio: (**a**) Alloy R-70 (third measurement), and (**b**) alloy R-90 (third measurement).

It can be seen from the graphs below (Figure 6 and Figure 7) that no stress occurs in the material at the beginning of solidification. As solidification continues and the temperature decreases, a crystalline structure begins to form, and deformation occurs. The casting begins to shrink, which is reflected in the increase of the tensile force recorded by the load cell.

The first and fourth measurements of the commercial grade alloy are characterized by a smooth force curve without hot tear detection. Figure 6a shows the graph with the courses of temperature, force and its derivatives of the fourth measurement. Hot tear occurrence was detected on the force curve during the second and third measurements. In both cases, there was no complete rupture of the arm, and a subsequent increase in force was recorded.

When using a 20% proportion of returnable material (R-20 alloy) in the batch, no hot tear was formed on the through-arm only in the third measurement. In the other three measurements, the formation of hot tears was detected, and after their propagation, a repeated increase in force (first measurement) and stabilization of the force was recorded, when the curve maintained a constant value (second and fourth measurements).

At the equilibrium batch ratio (R-50 alloy), the formation of a severe hot tear was recorded in the first and fourth measurements, which led to complete tearing off of the arm. Conversely, no hot tear formation was detected in the third measurement. The graph (Figure 6b) shows the course of the second measurement. It is characterized by the formation of a hot tear. It can be seen from the course of the curve that the arm did not rupture completely, and 80 s after the formation of the hot tear, its propagation was terminated by repeatedly increasing the rate of load. Table 5, Table 6 and Table 7 present detailed values of each measurement for the commercial grade alloy, R-20 and R-50 alloys obtained in the qualitative evaluation.

In the case of alloys with a predominant content of returnable material in the batch, not a single measurement was recorded without the occurrence of hot tearing. In the case of an alloy with a 70% content of returnable material, in two cases, the arm was completely torn off due to the formation a hot tear (third and fourth measurements). The graph in Figure 7a clearly shows that the tearing off of the arm during the third measurement did not occur immediately, but only after 6 s of hot tear propagation. With 80% returnable material in the batch, complete tearing off of the arm occurred in three cases. In the first measurement, the alloy was still able to stop the hot tear propagation with the subsequent re-increase of force.

The alloy with the highest content of returnable material (R-90) showed the worst results. The arm was torn off in all measurements (Figure 7b). The detailed values of each measurement for alloys with higher contents of returnable material (R-70, R-80, R-90) obtained in qualitative evaluation are given in Table 8, Table 9 and Table 10.

Table 11 shows the solidification interval of experimental alloys, which significantly affects the overall susceptibility of the alloy to hot tearing. Increase of returnable material in the batch led to a change in wt.% of some elements, especially Fe, Si and Cu (Table 1 and Table 2). Elements Si and Cu can affect the width of the solidification interval and thus also the susceptibility of the alloy to hot tearing [17,18,19]. From the temperatures, it can be stated that due to the increase of the returnable material in the batch and thus the change in weight. % of some elements, there was no significant change in the width of the solidification interval.

### 3.3. Fracture Surfaces and Tear Profile

**Figure 8:**
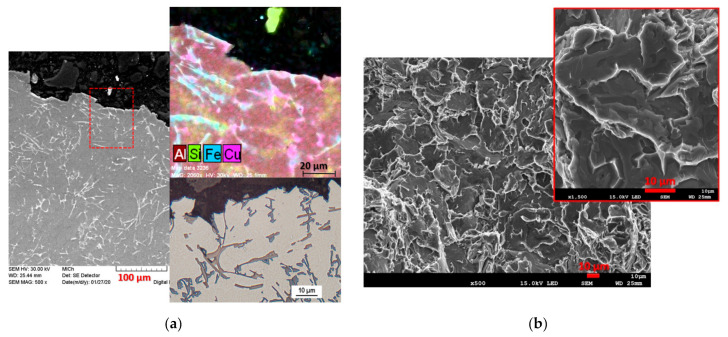
Evaluation of hot tear formation and propagation for alloy R-50, OM (optical microscope), SEM (scanning electron microscope). (**a**) Hot tear profile, (**b**) fracture surface.

The first alloy evaluated is R-50, which in the quantitative evaluation showed a moderate HTS (for HTI3) and in the qualitative evaluation there were two arm separations (Table 7). The hot tear profile of the R-50 alloy clearly shows that the weakest regions for hot tear propagation were the iron phases in acicular morphology (Figure 8a). The sharp ends of the iron phase acicular formations acted as a critical point for the formation of micro-tears. Due to the fragility of the phases, conditions were created for further hot tear propagation of a transcrystalline, but also with an inter-crystalline nature—mixed hot tear propagation (fracture). When looking at the arm fracture surface, brittle fracture predominates—failure by cleavage of iron-based intermetallic phases (Figure 8b).

**Figure 9:**
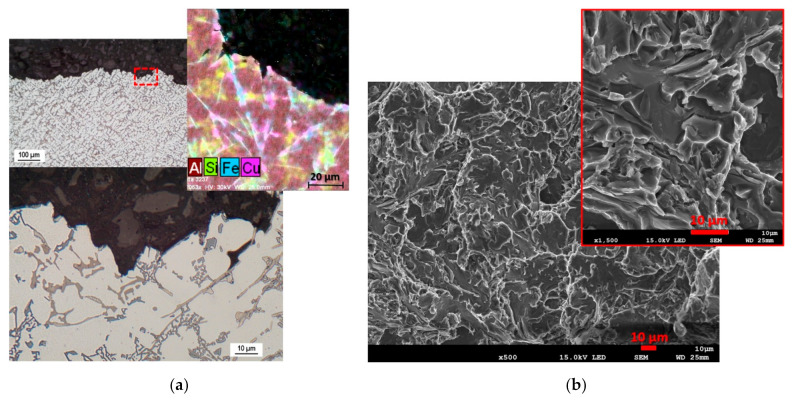
Evaluation of hot tear formation and propagation for alloy R-70, OM, SEM. (**a**) Hot tear profile, (**b**) fracture surface.

In the case of the alloy with a 70% content of returnable material (high HTS according to HTI_3_, Figure 5), the transcrystalline hot tear propagation mechanism is the main component of the hot tear profile shown in Figure 9a. Regions of closed micro-tears with a transcrystalline mechanism can be observed in the image of the hot tear profile. The fracture surface is, similarly to the alloy, characterized by brittle fracture—failure by cleaving thicker plates of iron phases (Figure 9b), which could have the greatest effect on the two detached arms in the qualitative evaluation, and on the immediate arm separation in the fourth measurement (Table 8).

**Figure 10:**
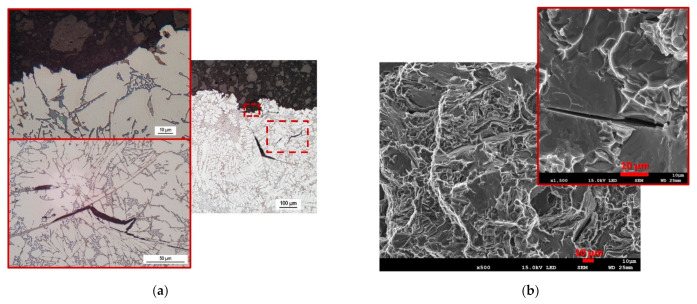
Evaluation of hot tear formation and propagation for alloy R-90, OM, SEM. (**a**) Hot tear profile, (**b**) fracture surface.

**Figure 11:**
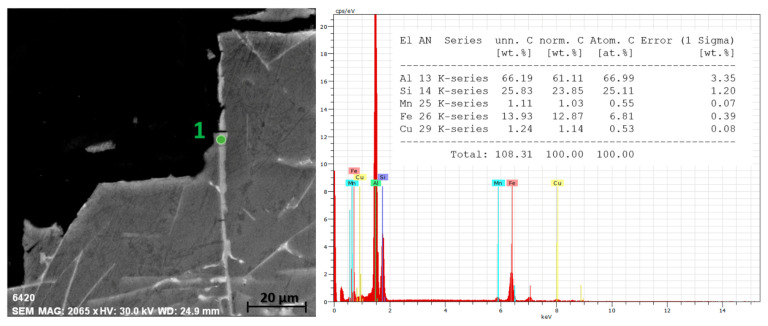
EDX (energy dispersive X-ray) analysis of the iron phase Al_5_FeSi in tear profile for alloy R-90, SEM.

The R-90 alloy with the largest content of returnable material reached the worst results in the qualitative test, when arms were torn off all cases (in three cases there was immediate tear-off, Table 10). The hot tear profile shows transcrystalline propagation of hot tears in brittle acicular formations of iron phases and a large occurrence of closed micro-tears in the region of the fracture profile (Figure 10a), which led to a very high susceptibility to hot tearing (Figure 5). The brittle fracture on the R-90 alloy fracture surface is also characterized by a significant inter-phase failure at the boundary between the iron phase plate and the matrix (Figure 10b). Figure 11 shows EDX analysis of the iron phase Al_5_FeSi in acicular morphology, which served as a stress concentrator and a suitable site for transcrystalline hot tear propagation.

## 4. Discussion

The above results prove that the negative effect of returnable material in the investigated alloy started to manifest itself already when it increased to a 20% content in the batch. The difference between the resulting values of the HTS index and qualitative evaluation was not significant when compared to the commercial grade alloy. It can be stated that the alloy with an equilibrium proportion of the commercial grade alloy and returnable material (R-50) was just beyond the permissible limit (HTS index—slight hot tearing susceptibility, two torn-off arms in qualitative evaluation).

It can be stated from Table 5, Table 6, Table 7, Table 8, Table 9 and Table 10, just like in the case of HTS evaluation, that with increasing content of returnable material in the batch, the hot tearing susceptibility visibly deteriorates even when using qualitative evaluation. The hot tears formed on alloys with no or low content of returnable material (commercial grade alloy, R-20 alloy) are characterized by a low hot tear formation temperature and a later time of their formation. Hot tearing temperatures for these alloys ranged from 380 to 467 °C, which represents temperatures lower than the temperatures of the alloys solidus (Table 11).

With the increasing content of returnable material in the batch, the hot tear formation shifted to times closer to the beginning of solidification of the alloy and thus to higher temperatures. Hot tears formed in alloys with a return material content of 70% and higher in the batch are already characterized by hot tear formation temperatures in the solidification interval (Table 11). For comparison, the hot tear formation time for the alloy with the highest content of returnable material in the batch was in the range of 3 to 7 s, while for the commercial grade alloy, R-20 alloy, the hot tear formation ranged from 9 to 15 s from the beginning of applying the load.

Due to the increase of the returnable material in the batch, there was a change in the wt.% of individual elements (Table 1 and Table 2). The most significant change occurred in the elements Si, Cu and Fe, while these elements can significantly contribute to the susceptibility of the alloy to hot tearing. However, the change of wt.% Si and Cu in experimental alloys were not large enough to significantly affect the solidification interval of experimental alloys (Table 11), which is directly involved in changing the susceptibility of the alloy to hot tearing.

Gradual increase in wt.% Fe with an increase in returnable material in the batch proved to be a major factor influencing the formation of hot tearing in experimental alloys. In the case of alloys with a high content of returnable material in the batch, an increased Fe content was fully manifested and thus an excessive occurrence of iron-based intermetallic phases was observed. The resulting Al_5_FeSi phase plates are primarily formed prior to the solidification of the eutectic, thereby reducing the deposition of the melt to critical sites in the casting in order to compensate for/limit the emerging hot tears and their further propagation [32]. The Al_5_FeSi phase plates (in the acicular cut plane) probably acted as a critical point for the formation of micro-tears and subsequently created an ideal place for their propagation. Brittle fracture predominated on all observed fracture surfaces, indicating failure by cleavage of iron-based intermetallic phases [20].

## 5. Conclusions

The study confirmed the effect of increasing the sensitivity of the AlSi9Cu3 alloy due to the increase in the returnable material content in the batch. The returnable material in the batch led to an increase in the hot tearing susceptibility rate from low (HTI_3_) for the commercial purity alloy (without returnable material in the batch) to a very high susceptibility of the alloy with a 90% content of returnable material. Metallographic evaluation shows that the main role in increasing the hot tearing susceptibility was played by the iron-based intermetallic phase Al_5_FeSi, the presence of which is characteristic mainly for the microstructure of alloys with a 50% and higher content of returnable material in the batch. 

It can be stated based on the results of HTS evaluation and qualitative evaluation that alloys with a returnable material content of about 20% in the batch can be used for the production of shape-demanding castings. Conversely, the use of alloys with a returnable material content above 50% can be problematic for the production of shape-complex castings in terms of hot tearing and are rather recommended for the production of shape-simple and less loaded castings.

## Figures and Tables

**Table 1:** Chemical composition of AlSi9Cu3 alloy by standard and AlSi9Cu3 commercial purity alloy (wt.%).

Elements	Si	Cu	Fe	Mg	Ti	Mn	Cr	Ni
AlSi9Cu3 (Commercial purity)	9.563	2.206	1.081	0.426	0.038	0.184	0.027	0.092

**Table 2:** Chemical composition of returnable materials of AlSi9Cu3 alloy and newly formed experimental alloys AlSi9Cu3 alloy (wt.%).

Elements	Si	Cu	Fe	Mg	Ti	Mn	Cr	Ni
**Returnable**	9.294	2.074	1.674	0.348	0.034	0.184	0.113	0.129
**R-20**	9.507	2.197	1.294	0.391	0.035	0.231	0.049	0.122
**R-50**	9.418	2.173	1.419	0.361	0.033	0.223	0.072	0.134
**R-70**	9.355	2.02	1.569	0.344	0.031	0.209	0.112	0.108
**R-80**	9.345	2.084	1.617	0.358	0.032	0.206	0.101	0.156
**R-90**	9.382	2.043	1.643	0.357	0.032	0.199	0.106	0.127

**Table 3:** Evaluation system AL (arm length) and TP (tear position) for HTI_2_.

Arm Length Coefficient	AL	Tear Position Coefficient	TP
**Arm 1 (64.5 mm)**	1	**Lower part of arm (sprue end)**	1
**Arm 2 (104.5 mm)**	2	**Middle part of the shoulder**	3
**Arm 3 (124.5 mm)**	3	**Upper part of arm (ball end)**	2
**Arm 4 (184.5 mm)**	4		

**Table 4:** Hot tearing susceptibility (HTS) intervals [16].

HT Index	<0.5	0.5–1.25	1.25–2.25	2.25–3.5	>3.5
**HTS (Susceptibility)**	Minimal	Low	Moderate	High	Very high

**Table 5:** AlSi9Cu3 Commercial purity.

No.	Hot Tear Initiation				End Hot Tear Propagation		
	Temperature (°C)	Time (s)	Load (N)	Load Force Ratio (N/s)	Temperature (°C)	Time (s)	Type of End Hot Tear Propagation
**1.**	No hot tear		max. 1954	max. 115.7	No hot tear		
**2.**	467	10	227	24.3	308	88	Increase of load
**3.**	380	14	582	50.2	226	105	Increase of load
**4.**	No hot tear		max. 1590	max. 96.4	No hot tear		

**Table 6:** R-20 alloy.

No.	Hot Tear Initiation				End Hot Tear Propagation		
	Temperature (°C)	Time (s)	Load (N)	Load Force Ratio (N/s)	Temperature (°C)	Time (s)	Type of End Hot Tear Propagation
**1.**	451	9	486	54.6	427	39	Increase of load
**2.**	396	15	712	43.2	396	56	Stabilization of load
**3.**	No hot tear		max. 1154	max. 89.5	No hot tear		
**4.**	413	12	387	35.2	344	81	Stabilization of load

**Table 7:** R-50 alloy.

No.	Hot Tear Initiation				End Hot Tear Propagation		
	Temperature (°C)	Time (s)	Load (N)	Load Force Ratio (N/s)	Temperature (°C)	Time (s)	Type of End Hot Tear Propagation
**1.**	456	10	407	38.7	426	18	Arm separation
**2.**	443	11	242	24.7	252	80	Increase of load
**3.**	No hot tear		max. 672	max. 54.6	No hot tear		
**4.**	509	9	661	61.2	361	47	Arm separation

**Table 8:** R-70 alloy.

No.	Hot Tear Initiation				End Hot Tear Propagation		
	Temperature (°C)	Time (s)	Load (N)	Load Force Ratio (N/s)	Temperature (°C)	Time (s)	Type of End Hot Tear Propagation
**1.**	541	3	242	37.6	532	6	Increase of load
**2.**	474	11	767	80.1	465	30	Stabilization of load
**3.**	478	10	143	21.2	462	16	Arm separation
**4.**	521	6	97	25.9	Immediate arm separation		

**Table 9:** R-80 alloy.

No.	Hot Tear Initiation				End Hot Tear Propagation		
	Temperature (°C)	Time (s)	Load (N)	Load Force Ratio (N/s)	Temperature (°C)	Time (s)	Type of End Hot Tear Propagation
**1.**	470	11	584	63.9	341	41	Increase of load
**2.**	505	9	136	20	461	16	Arm separation
**3.**	532	5	74	11.5	Immediate arm separation		
**4.**	551	4	19	6.7	328	50	Arm separation

**Table 10:** R-90 alloy.

No.	Hot Tear Initiation				End Hot Tear Propagation		
	Temperature (°C)	Time (s)	Load (N)	Load Force Ratio (N/s)	Temperature (°C)	Time (s)	Type of End Hot Tear Propagation
**1.**	563	3	445	40.7	Immediate arm separation		
**2.**	543	5	374	32.4	473	14	Arm separation
**3.**	537	6	54	5	Immediate arm separation		
**4.**	514	7	254	17.6	Immediate arm separation		

**Table 11:** Solidification interval (SI) of experimental AlSi9Cu3 alloys.

Alloy	Commercial Purity	R-20	R-50	R-70	R-80	R-90
**SI (°C)**	631 to 479	630 to 474	631 to 477	633 to 476	628 to 473	632 to 476

## Data Availability

Data available on request. The data presented in this study are available on request from the corresponding author.
